# Testis-specific serine kinase protein family in male fertility and as targets for non-hormonal male contraception^†^

**DOI:** 10.1093/biolre/ioaa064

**Published:** 2020-04-27

**Authors:** Ana M Salicioni, María G Gervasi, Julian Sosnik, Darya A Tourzani, Saman Nayyab, Diego A Caraballo, Pablo E Visconti

**Affiliations:** 1 Department of Veterinary and Animal Sciences, University of Massachusetts-Amherst, Integrated Sciences Building 427S, 661 North Pleasant Street, Amherst MA 01003, USA; 2 Molecular and Cellular Biology Graduate Program, University of Massachusetts, Amherst, MA, USA; 3 Department of Biology, University of Massachusetts, Boston, MA, USA; 4 Biotechnology Training Program, University of Massachusetts, Amherst, MA, USA; 5 IFIBYNE-CONICET, Department of Physiology, Molecular and Cellular Biology, University of Buenos Aires, Buenos Aires, Argentina

**Keywords:** TSSK, testis-specific, kinases, sperm, spermatogenesis, fertilization, non-hormonal male contraceptive, evolution, intronless gene, pseudogenization

## Abstract

Male contraception is a very active area of research. Several hormonal agents have entered clinical trials, while potential non-hormonal targets have been brought to light more recently and are at earlier stages of development. The general strategy is to target genes along the molecular pathways of sperm production, maturation, or function, and it is predicted that these novel approaches will hopefully lead to more selective male contraceptive compounds with a decreased side effect burden. Protein kinases are known to play a major role in signaling events associated with sperm differentiation and function. In this review, we focus our analysis on the testis-specific serine kinase (TSSK) protein family. We have previously shown that members of the family of TSSKs are postmeiotically expressed in male germ cells and in mature mammalian sperm. The restricted postmeiotic expression of TSSKs as well as the importance of phosphorylation in signaling processes strongly suggests that TSSKs have an important role in germ cell differentiation and/or sperm function. This prediction has been supported by the reported sterile phenotype of the *Tssk6* knockout (KO) mice and of the double *Tssk1* and *Tssk2* KO mice and by the male subfertile phenotype observed in a *Tssk4* KO mouse model.

## Teaser

The established role of TSSKs in spermiogenesis and in male fertility, together with their unique kinase features, strongly supports this family of protein kinases as a very promising drug discovery target for development of a non-hormonal male contraceptive.

## Introduction

The ability to control fertility through the accessibility to effective contraceptive methods is an indispensable component of preventive health. Contraceptive methods are vital for individuals and families; they also play an essential role in population dynamics and deserve a central place both in the basic science and in reproductive medicine. Research on newly developed male and female contraceptive methods, as well as refinement of existing ones, has been supported by the reproductive medicine platform of the NICHD since 1969 [1], more recently implemented through the scientific agenda of the Contraceptive Development Program. Making this a top-priority health matter should be conducive to reducing the burden of unintended pregnancies and to ensuring that every child is wanted and reaches their full potential. Despite the availability of a range of contraceptive methods, the unintended pregnancy rate in the USA is ~ 45% [2] and over 50% (~1 000 000/day) worldwide with a high percentage of them terminated by abortion. Thus, there is clearly an unmet need for contraceptive methods for men and women to better fit their family planning goals and which take into consideration their different ethnic, cultural, and religious values [3, 4].

One goal of ongoing research is to develop a male oral contraceptive that could be taken as a pill. Recent acceptability studies indicate that a high percentage of men would be willing to use a male contraceptive pill [5, 6]. In addition, women organizations are increasingly demanding that men take a more active role in family planning [7], in order to increase couples’ success in achieving their desired timing and number of children. However, the availability and use of reversible male-based contraceptive methods is rather limited, including male condoms and withdrawal, which show rate failures of up to 20% [8]. Among the hormonal approaches for a male pill, testosterone promotes suppression of spermatogenesis by reduction in the production of gonadotropins and has shown very promising results in clinical trials [9, 10]. However, this approach has been hindered by the lack of a safe, effective oral androgen component. Current development includes long-acting injectables and transdermal gels. Novel androgens that may have both androgenic and progestational activities are also part of the search for a single-agent male hormonal contraceptive method [11, 12]. At the moment, however, the lack of long-term studies has precluded the development of a marketable product. Given the general safety and efficacy requirements posed on every contraceptive approach, as well as the anticipated undesired side effects and other issues related to the complete reversibility of fertility, there is an unquestionable need for alternative approaches for controlling male fertility.

During the past decade, several non-hormonal targets for development of a male contraceptive have been brought to light and are the focus of active research both in the academic setting and in the pharmaceutical industry [13–16]). The rationale behind a non-hormonal male contraceptive approach is to circumvent the hypothalamic–pituitary axis, thus avoiding some side effects associated with the use of hormones. It is predicted that these novel methods will likely lead to more selective contraceptive compounds with superior side effect profiles. Non-hormonal contraceptives may be more easily dosed orally than most steroid preparations. In addition, a recent consumer research study sponsored by the Male Contraceptive Initiative shows that sexually active men (ages 18–44) are twice as likely to prefer a non-hormonal (80%) to a hormonal (38%) method [17]. The strategy is to target genes along the pathways of sperm production, maturation, or function.

Spermatogenesis is the process by which morphologically differentiated spermatozoa are produced in the testis and takes place in three main phases: (1) proliferative or spermatogonial phase in which spermatogonia undergo mitotic divisions and generate a pool of spermatocytes; (2) meiotic phase in which haploid spermatids are generated; and (3) spermiogenesis in which a round spermatid is differentiated into a spermatozoon. The molecular mechanisms that regulate the first two phases are relatively well known [18], while molecular events that take place during spermiogenesis are poorly understood. Knowing the importance of phosphorylation events in the regulation of cellular signaling processes and differentiation, it is not surprising that several protein kinases have been shown to be key players in spermatogenesis. Interestingly, a few of these kinases have testis-specific splicing variants, with the catalytic subunit of PKA and the casein kinase II alpha’ (*CKIIα’)* being two of the most relevant cases. Null mice for these enzymes are sterile. In the case of PKA, null mutants of the sperm-specific catalytic subunit (Cα2) are sterile and have deficient sperm motility and capacitation [19]. The sterile phenotype observed in the *CKIIα’* knockout mice is due to defective spermiogenesis and a globozoospermic phenotype [20]. Although many of the abovementioned kinases could be potential targets for male contraception, they have the same catalytic domain as their somatic counterparts, thus limiting the search for inhibitors to molecules targeted to domains that play a role in protein–protein interactions. Interestingly, HIPK4, a member of the homeodomain-interacting protein kinase (HIPK) subfamily, is predominantly expressed in the testis and has been shown to play a role in spermatogenesis. HIPK4 is a dual-specificity kinase with a restricted localization to round and early elongating spermatids in mouse testis; localization in human testis tissue has also been confirmed [21]. *HIPK4* knockout mice are infertile in vivo and by in vitro fertilization (IVF), although they can produce viable progeny by intracytoplasmic sperm injection (ICSI). The oligoasthenoteratozoospermia phenotype observed in *HIPK4*-null mice is indicative of aberrant F-actin dynamics, which in turn would affect the acrosome differentiation in the elongating spermatids and lead to abnormal sperm heads in mature sperm. Overall, HIPK4 function in mouse spermatogenesis suggests this kinase as a novel, potential target for male contraception.

Other testis kinases belong to the testis-specific serine kinase (TSSK) **family**. The TSSK protein family is composed of six members, TSSK1 through TSSK6 forming a branch within the calcium/calmodulin-dependent protein kinases (CAMK) superfamily; *TSSK5* has been described as a pseudogene in humans and other primates [22], and mouse TSSK5 might not perform as an active kinase [23]. This review will focus on the highly conserved TSSK kinases, their role in sperm physiology, and their potential use as drug discovery targets for non-hormonal male contraceptives.

## Discovery and cloning of TSSKs

In 1994, the group led by Ziemiecki, using a degenerate primer strategy in searching for novel kinases, reported the cloning of a mouse testis-specific kinase and named it *Tsk1* [24]. By sequence homology, they determined that *Tsk1* shared homology to the human *rac* protein kinase b and a group of serine/threonine kinases from yeast. Soon after, Buck and collaborators identified a region in the mouse chromosome 16 syntenic to the DiGeorge syndrome minimal region in human chromosome 22 [25]. This region contained genes encoding for several novel proteins including a member of the TSSK family with high homology to the putative testicular kinase reported by Bielke and colleagues [24]. In a second manuscript, Ziemiecki and his lab found a second member of this family and renamed them to Tssk1 and Tssk2 (formerly named Tsk1 and Tsk2, see Table 1 for current nomenclature). In this work, the authors showed that both Tssk1 and Tssk2 were capable of phosphorylating TSKS (testis-specific kinase substrate), a ~65 kDa protein of unknown identity. They also showed for the first time that all these proteins (TSSK1, TSSK2, and TSKS) were expressed postmeiotically during spermatogenesis and showed experimentally that both Tssk1 and Tssk2 behaved as serine kinases [26]. In a later work, Nayak and collaborators [27] confirmed these observations and proposed an initial association of Tssk1 with cells in meiotic metaphase and a later association of Tssk2 with tail-like structures in the lumen of the seminiferous tubule.

**Table 1:** Current nomenclature and genome-annotated designations for human and mouse TSSKs (Sources: Uniprot [83], ENSEMBL [84], and HUGO Gene Nomenclature Committee (HGNC) [85] databases).

Human							Mouse		
Official symbol	Protein aliases	Gene name	Chromosome location	Predicted kinase activity	Official symbol	Protein aliases	Gene name	Chromosome location	Predicted kinase activity
TSSK1		TSSK1A					Tsk1		
		SPOGA1				TSK-1	TSK-1		
		STK22A				TSSK-1	Tssk		
	N/A	TSSK1	22q11.21	NO	TSSK1	Tsk1	Tssk1	16, 11.09 cM	YES
		TSSK7P				Stk22a	Stk22a		
		TSSK1AP					Tssk1b		
		Pseudogene							
		TSSK1B							
	TSSK1	TSSK1							
	TSK1	FKSG81							
	TSK-1	SPOGA4	5q22.2	YES					
	TSSK-1	SPOGA1							
	STK22A	STK22D							
		STK22A							
TSSK2	DiGeorge syndrome protein G	TSSK2				Tssk2	Tsk2		
	(DGS-G)	DGS-G				TSSK2	Tssk2		
	TSK-2	SPOGA2	22q11.21	YES	TSSK2	TSK-2	Stk22b	16, 11.09 cM	YES
	TSK2	STK22B				TSK2	SPOGA2		
	TSSK-2	FLJ38613				TSSK-2	DGS-G		
	TSSK2					Tsk2			
	SPOGA2					Stk22b			
	STK22B								
TSSK3	SPOGA3					Tssk3	Stk22c		
	STK22C	SPOGA3				TSK-3	Stk22d		
	TSK3	STK22C				TSSK-3	Tssk3		
	TSSK-3		1p35.1	YES	TSSK3		Tsk3	4, 63.26 cM	YES
	TSK-3	TSSK3				Stk22c	Tssk-3		
	TSSK3					Stk22d	1700014N07Rik		
							4930594I21Rik		
TSSK4	TSSK4					Tssk4			
	TSSK-4	TSK4					Tssk4		
	TSK-4	TSSK4				Tsk4	Tssk5	14, 28.19 cM	YES
	TSK4	TSSK5	14q12	YES	TSSK4	TSK-4	1700020B19Rik		
	TSSK5	STK22E				TSSK-4	4933424F08Rik		
	STK22E	C14orf20							
	C14orf20								
TSSK5		TSSK5P							
		TSSK5P1				Tssk5	Tssk5	15, 35.79 cM	NO
	N/A	TSSK5P2	8q24.3	NO	TSSK5	TSK-5	1700091F14Rik		
		TSSKps1				TSSK-5			
		Pseudogene				Tsk5			
TSSK6	TSSK6								
	TSK-6								
	TSSK-6	TSSK6				Sstk			
	SSTK	SSTK				Tsk6	Tssk6		
	TSSK4	TSSK4	19p13.11	YES	TSSK6	Tssk6	Sstk	8, 34.05 cM	YES
	CT72	CT72				TSK-6			
	FKSG82	FKSG82				TSSK-6			
	FLJ24002	FLJ24002							

Later reports expanded this family of kinases with the cloning of *Tssk3* in mouse [28, 29]. Tssk3, as well as its human homolog TSSK3, are also exclusively expressed postmeiotically in testicular germ cells [29]. Both genes were mapped using fluorescent in situ hybridization to syntenic regions of human and mouse chromosomes 1q34-q35 and 4E1, respectively [29]. Human *TSSK1* and *TSSK2* were cloned and shown to be testis-specific [30]. Using qRT-PCR with RNA from human tissues as templates, this latter study also showed that transcripts for human TSSK3 as well as for a fourth member of the TSSK family were also testis-specific. Originally named TSSK4 [30], this kinase was later renamed SSTK and it is now known as TSSK6 (see Table 1). A fifth TSSK member, originally named TSSK5 and now known as *TSSK4*, was cloned in humans by Chen and his colleagues in 2005 [31]. Its mouse ortholog was identified as presenting different splicing variants [32], which was a significant finding since all other Tssk mouse transcripts are intronless. Mutations in the *TSSK4* gene were recently linked to impaired spermatogenesis in Chinese infertile men [33].

Cloning of Tssk homologs has also been reported in bull [34] and in the marine bivalve mollusk *Argopecten purpuratus* [35]. Marcello et al. [36] reported that three TSSK orthologs are expressed in the nematode *Caenorhabditis elegans* and that deletion mutants carry male gametes with different degrees of abnormality. In 2015, five members of the TSSK family were cloned and characterized in a Banna mini-pig inbred line [37]. Very recently, a TSSK1-like gene was cloned and characterized in the Pacific abalone species, *Haliotis discus hannai* [38], a commercially important marine gastropod. RT-PCR analysis of somatic and gonadic tissues from both mature female and male abalones showed a significant expression of *TSSK1-like* transcripts in abalone testis and, at lower levels, in mature sperm. This finding, along with analysis of abalone TSSK1-like amino acid sequences, suggested structural orthology of abalone TSSK1-like to human TSSK1 and other members of the TSSK family, especially in relation to its catalytic kinase domain.

## Evolutionary considerations

Studying the evolution and function of testicular genes, in particular structure–function differences between mice and humans, is very relevant not only to further our understanding of spermatogenesis but also to move forward with our knowledge on male infertility [39]. The TSSK family of proteins is a highly conserved group of protein kinases, present in a wide variety of organisms. Its origin can be traced back to the ancestor of amniotes (between 380 and 316 MYA), but a significant increase in copy number via duplication events has taken place in the ancestor of mammals [40]. In a given species, several paralog members of these genes are found and display a high level of homology. Importantly, the degree of similarity between orthologs in different species is also high [26, 25]. In a very elegant study, Shang et al. [41] analyzed sequences of genes encoding for TSSK enzymes in different species and proposed that TSSK1 and TSSK2 appeared in the common ancestor of all mammalian species (monotremes, marsupials, and placental mammals). These two genes may have originated by a RNA-based retroposition event, from an unknown parental gene. The newest gene of this kinase branch, *TSSK1B*, was found in new- and old-world monkeys, apes, and humans. According to Shang and colleagues [41], positive selection during evolution would explain a higher percentage of sequence mutations in the C-terminal domain of TSSK1/TSSK1B, in comparison to TSSK2, which might be associated to differential preference for protein partners and substrates.

**Figure 1:**
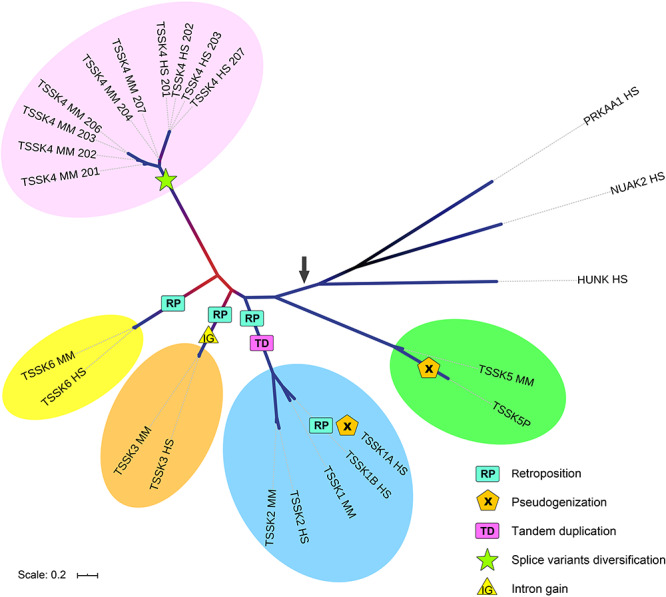
Bayesian phylogenetic tree showing evolutionary relationships among human and mouse TSSKs. A first analysis was run integrating over a predetermined set of fixed rate matrices, where the Jones model depicted the highest support. The definitive analysis was performed fixing the Jones model for 1 × 10^7^ Markov Chain Monte Carlo (MCMC) generations, sampling every 1000 generations. The first 25% MCMC generations were discarded as burnin. The estimated sample size (ESS) was below 0.01 by 11.45% of the MCMC chain, where it showed as well a stationary trace distribution. Protein sequences of phylogenetically related CAMK human kinases HUNK, NUAK2, and PRKAA1 were included, fixing the latter as outgroup. Branch color depicts node support being red/dark purple/blue for [0.6–0.7]/(0.7–0.8]/(0.8–1.0) posterior probability values. The black arrow indicates the origin of the ancestral TSSK. Intron gains, origin of splice variants, tandem duplications, paralog pseudogenization, and retroposition events are mapped in the phylogeny.

To gain insight into evolution of human and mouse TSSKs, we performed a Bayesian phylogenetic analysis based on protein sequences. This analysis aimed to evaluate the homology of human and mouse TSSKs, as well as to test the basal position of TSSK5 which may resemble the ancestral form of TSSKs [41]. To test the monophyly of TSSKs, as well as to polarize the evolutionary tree, we included protein sequences of human kinases which are phylogenetically close within the CAMK superfamily [42], HUNK, NUAK2, and PRKAA1, fixing the latter as outgroup. Protein sequences were aligned using Clustal Omega at the EMBL-EBI website [43]. Phylogenetic analyses were run in MrBayes 3.2.6 [44] on the CIPRES Science Gateway [45]. This Bayesian phylogenetic analysis revealed that *TSSK5* is the most basally splitting *TSSK* (Figure 1), in agreement with the hypothesis which states that this paralog may resemble the parental gene of the family [41]. All paralogs exhibited comparable levels of differentiation, except for the recently duplicated *TSSK1B* and *TSSK2* which depict lower levels of divergence reflected by shorter branch lengths (Figure 1). Divergence levels were substantially lower between human and mouse orthologs (e.g., TSSK2 HS and TSSK 2 MM) than between paralogs (e.g., TSSK2 HS and TSSK3 HS). The existence of splicing variants in TSSK4 conferred an increment in protein variability, especially in mouse, where we found two isoforms which depicted considerably divergent C-terminal domains.

As mentioned above, the *TSSK5* gene in primates underwent pseudogenization. The human gene *TSSK5P* (ENSG00000227473) is a unitary pseudogene. This gene was lost in primates, after the Platyrrhini (new-world monkeys) and Catarrhini (apes and old-world monkeys) split (between 42.9 and 30.5 MYA, [22]). The first 101 codons of the human gene can be translated, and these correspond to the similar first 101 codons of the coding sequence of the mouse gene, but it is interrupted by a premature stop codon. The protein kinase domain is relatively long (ca 250 residues) in all TSSKs, so the resulting peptide represents less than half of the functional domain. Moreover, this fragment only accounts for the first two exons, followed by five introns, so it would certainly trigger the nonsense-mediated decay pathway [46]. Hence, it is highly unlikely that any protein product would be produced from this gene. The TSSK5P transcript is expressed in several tissues, but unlike other TSSKs (including TSSK5, its paralog present in new-world monkeys and non-primate mammals), it is not primarily transcribed in the testis but in the brain and cerebellum [47]. After loss of function, this gene underwent regulatory alterations with no selective effect, which led to a shift in tissue-specific transcription. Taking into account that *TSSK5* is a functional gene conserved since the emergence of amniotes, our analysis indicates that in humans (and other primates) this gene may have been functionally replaced by a paralog. Possibly, the emergence of the primate-specific TSSK1B, a new copy of TSSK located in a different regulatory background, could account for the replacement of TSSK5P in this group.

## TSSKs expression and localization

**Figure 2:**
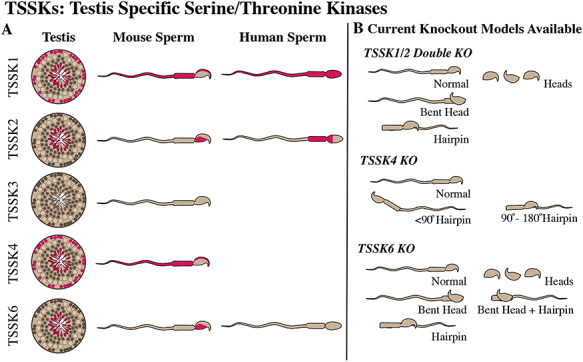
(A) Graphic representation of the localization of TSSK1, TSSK2, TSSK3, TSSK4, and TSSK6 protein kinases based on previous work from our laboratory [23]. Localization in mouse testis (left), mouse sperm (middle), and human sperm (right). (B) Current Tssk knockout mouse models available, and morphological sperm defects associated with each *Tssk* knockout [52, 40, 61].

Our laboratory performed a comprehensive analysis of the expression and localization patterns of TSSK family members in mouse and human sperm [23]. In this study, using quantitative PCR, we confirmed that the transcripts of *Tssk1*, *Tssk2*, *Tssk3*, *Tssk4*, and *Tssk6* are testis-specific and postmeiotically expressed in mouse germ cells. In addition, using validated antibodies, we characterized and identified the subcellular localization of these kinases during spermatogenesis and in mature sperm (Figure 2), confirming and expanding observations reported by other authors [26, 29, 30, 32, 48, 40]). As schematically shown in Figure 2A, we found that Tssk1, Tssk2, Tssk4, and Tssk6 were all postmeiotically expressed in spermatids. However, while Tssk2 and Tssk6 proteins were detected exclusively in spermatids, fluorescence immunostaining for Tssk1 and Tssk4 was also detected close to the basal membrane, most probably in spermatogonia. These data suggested that Tssk1 and Tssk4 might be expressed in other spermatogenic germ cells that had not completed meiosis. Alternatively, we reasoned that the antibodies against Tssk1 and Tssk4 were cross-reacting, non-specifically, with some other protein(s) when used for immunostaining [23]. In mouse sperm, Tssk1 and Tssk4 were localized in the anterior head, seemingly in the acrosome, as well as in the sperm flagellum. Our data showed that mouse Tssk2 and Tssk6 are localized in a sperm head postacrosomal region, which is rich in polymerized actin [49]. It is interesting to note that *Tssk3* mRNA was detected in mouse spermatids, confirming earlier observations obtained by Northern blotting [29], yet we were unable to detect the Tssk3 protein either in mouse testis or in mature sperm using two separate anti-Tssk3 antibodies validated with recombinant proteins. TSSK1, TSSK2, and TSSK6 were also immunolocalized in human sperm and replicated a very similar pattern than the ones observed in mouse sperm. Although we were able to detect mouse Tssk4 both in mouse testis and in mature sperm, our anti-peptide antibody was not able to recognize human TSSK4. More recently, Wei et al. [50] confirmed the testis-exclusive, postmeiotic expression of mouse Tssk4 using a custom-made, polyclonal antibody. This anti-Tssk4 antibody was raised against a different C-terminal region than the antibody used in our analysis and was able to detect only one of the four expected endogenous Tssk4 isoforms in mouse testis, suggesting that only one Tssk4 variant is expressed in vivo. Overall, our study provided further support to the notion that TSSK family members display different cellular localizations, thus pointing to sub-functionalization rather than a redundant role for these protein kinases.

## In vitro biochemical characterization of TSSK kinases

Attempts have been made to understand the biochemical features of this family of kinases and to characterize its enzymatic activity. As part of the AMPK protein kinase family, TSSKs are required to be phosphorylated at a conserved threonine residue in the kinase domain, known as T-loop, in order to be active. Interestingly, it has been shown that AMPK and other related kinases are regulated by an upstream kinase active on the T-loop. However, neither TSSK1 nor TSSK2 or TSSK6 requires a second kinase and appears to undergo autophosphorylation in the T-loop threonine residue [51, 52]. In vitro, recombinant TSSK3 was shown to undergo autophosphorylation in the T-loop, and that mutation in its T-loop (threonine to alanine) resulted in TSSK3 inactivation [53]. Furthermore, these authors demonstrated that PDK1 could act as an upstream kinase in vitro, to phosphorylate this threonine residue (Thr168) and to subsequently activate TSSK3. More recently, Wei and colleagues reported that TSSK4 has a comparable autophosphorylation activity at Thr197 in the T-loop which is essential for its kinase activity [50].

At present, there is not much information about TSSKs in vivo substrates, and several groups have opted for in vitro approaches using recombinant proteins. TSSK1 and TSSK2 are able to interact with a 65-KDa protein known as testis-specific kinase substrate (TSKS), originally identified by yeast two-hybrid methodology using TSSK2 as bait [26]. In addition, human TSSK2 co-immunoprecipitates with and can phosphorylate human TSKS in vitro [30], and it has been shown that the TSSK2/TSKS kinase/substrate pair is colocalized in spermatid centrioles during flagellogenesis [48]. Interestingly, TSKS can be phosphorylated by both TSSK1 and TSSK2 [26] but not by TSSK3 [28].

In vitro, TSSK3 phosphorylates peptides containing an RRSSSV(Y) motif [53], which is not a suitable substrate for either TSSK1 or TSSK2. On the other hand, TSSK1 has been shown to phosphorylate the AMARA peptide (AMARAASAAALARRR), a commonly used AMPK substrate [54, 51], and it is predicted that all TSSKs will phosphorylate this substrate. Two peptides, Pep4 (PLSRTLSVSS) and Pep8 (AALVRQMSVAFFFK), have also been reported as suitable TSSK1 substrates for high-throughput screening (HTS) by luminescence detection [55]. TSSK6 is able to phosphorylate the GKGRGLSLARFAKK peptide from the myelin basic protein (MBP) sequence [52]. We have also used this MBP peptide for in vitro quantitative kinase activity assays and found that phosphorylation of the MBP (104–118) fragment (GKGAGLSLSRFSWGA) by TSSK2 is already significant at 5 μM peptide concentration, with a Km of ~ 10 μM for ATP. In addition, we determined that contrary to TSSK3 which requires Mn^2+^ for kinase activity [53], TSSK1 and TSSK2 have an absolute requirement for Mg^2+^ and are essentially not active in the presence of Mn^2+^ [23].

Using a yeast two-hybrid approach with the cAMP response element-binding protein (CREB; previously known as AMP-responsive transcription factor) as bait, Chen and coworkers found that TSSK4 can phosphorylate CREB and may stimulate the CRE/CREB pathway by phosphorylation of CREB at Ser-133 [31]. More recently, TSSK4 was proposed as one of the kinases involved in phosphorylation of outer dense fiber 2 (Odf2), an essential protein in the sperm flagellum [56]. Using a phospho-specific antibody, in vitro phosphorylation of Odf2 in Ser76 by Tssk4 was verified by co-transfection of Tssk4 and Odf2 serine/tyrosine/lysine-to-alanine point mutants in 293 T cells. In this study, further analysis of mouse sperm lysates by LC-MS/MS indicated that phosphorylation of Odf2-Ser76 by Tssk4 may occur in vivo, either directly or indirectly, providing mechanistic indication on the role of Tssk4 in sperm flagellum integrity and function. Another report, also through the use of a yeast two-hybrid system, reported that TSSK2 can phosphorylate SPAG16L, an axoneme protein necessary for flagellar movement. The TSSK2–SPAG16L interactions were proposed to occur via the WD repeats in TSSK2 C-terminal domain. Furthermore, SPAG16L null mice showed a significant reduction in TSSK2 expression in the testis [57]. Considering the respective localization of TSSK1 and TSSK2 ([23] and Figure 2) as well as the high homology between TSSK1 and TSSK2, it is not possible to rule out that SPAGL interacts with TSSK1. In addition to its putative interaction with SPAGL, TSSK1 was found to interact with the testis-specific phosphatase isoform PPP1CC2 [58]. These authors provided evidence indicating that PPP1CC2 interaction with TSSK1 is indirect, by forming a triad with TSKS, and proposed that this testicular kinase/phosphatase complex plays a critical role in normal development of sperm mitochondrial sheath during spermatogenesis.

TSSK6 (formerly known as SSTK, see Table 1) is one of the smallest kinases in the TSSK family and was shown to interact with heat shock proteins, HSP70, and HSP90 [52] in an association that would be required for TSSK6 activity. In a series of very elegant studies to further characterize this interaction, a novel co-chaperone, SSTK-interacting protein (SIP) was discovered via a yeast two-hybrid screen [59]. In this study, using two specific anti-SIP antibodies, SIP was found to be expressed only in mouse and human testis, in elongated spermatids, but not in mature sperm. Colocalizaton of SIP/TASCC (TSSK6-activating co-chaperone) and TSSK6 was detectable in the testis but not in ejaculated sperm, suggesting that SIP may not be needed once TSSK6 is a fully mature and active kinase. Furthermore, the authors provided evidence on SIP being a co-chaperone involved in the HSP90-dependent TSSK6 enzymatic activation. This association between SIP, TSSK6, and HSP90 appears to be indirect, possibly by SIP binding to HSP70 first, for later recruitment into the HSP90-TSSK6 complex, thus facilitating the conformational activation of TSSK6. The role of HSP90 in TSSK6 activation was later on expanded to include other members of the TSSK family. By co-expression in 293 T cells or COS7 cells, HSP90 was found to directly interact with TSSK1, TSSK2, TSSK4, and TSSK6, thus protecting these kinases against protein instability and proteasome-dependent degradation [60]. Interestingly, recombinant TSSK4 and TSSK6 catalytic activation was sensitive to pharmacological inhibition of HSP90, while recombinant TSSK1 and TSSK2 were unchanged. It is also important to note that, although recombinant TSSK3 appeared to be altered by HSP90 inhibition, the TSSK3–HSP90 direct interaction could not be demonstrated using this experimental design. To extend these observations, the authors established a primary cell culture model of mouse spermatids and were able to confirm that endogenous levels of TSSK2 and TSSK6 were significantly lowered by inhibition of HSP90. This HSP90-dependent response was specific for TSSKs, and not detected for other kinases such as GSK3β or the testis-specific linker histone H1T protein. Overall, these studies support the view that HSP90 has a specific role as a regulator of TSSK levels in germ cells, by stabilization and conformational maturation of these kinases, and by preventing them from ubiquitination and proteasomal degradation. In addition, the authors hypothesized that the differential response to HSP90 observed among the TSSKs is due to differences found among TSSKs C-terminal length and/or primary structure.

## In vivo analysis of TSSK kinases by knockout mouse models

The strongest evidence on the relevance of TSSK kinases in spermatogenesis and male fertility has been attained by generation of genetic knockout mouse models. The mouse *Tssk1* and *Tssk2* genes are located in tandem on chromosome 16, separated by an intergenic region of only 3.1 kb, making easier the deletion of both of these genes together by homologous recombination, which was accomplished independently by two groups [61, 40]. One of the groups reported that targeted deletion of *Tssk1* and *Tssk2* resulted in male chimeras carrying the mutant allele in spermatogenic cells, and that this allele was not transmitted to the offspring, indicating that the male infertility phenotype was due to haploinsufficiency [61]. The second group, using another mouse genetic background, generated a fertile *Tssk1/Tssk2* heterozygous mutant mouse line, which allowed them to obtain the *Tssk1* and *Tssk2* double knockout [40]. Furthermore, this group found that the male infertility phenotype resulting from lack of expression of Tssk1/Tssk2 was a consequence of developmental dysregulation and the collapsing of the mitochondrial sheet in late spermatids. Mechanistically, these authors hypothesized that Tssk1 and Tssk2 are involved in the functional transformation of the chromatoid body (CB), known to be involved in RNA storage and metabolism and is normally located in the cytoplasm of male germ cells. The CB would typically transition to a ring-shape structure in elongating spermatids in a complex containing Tssk1, Tssk2, and TSKS but was found severely disordered in testis tissue of *Tssk1* and *Tssk2* double knockout mice.

Recent work by Wang and coworkers has demonstrated that *Tssk4* knockout male mice exhibit a subfertility phenotype due to a significant decrease in sperm motility [62]. The authors observed that the sperm head morphology of *Tssk4* knockout male mice was normal, while the flagellum was bent, with obvious abnormalities in the midpiece–principal piece junction, which corresponds to a structure termed annulus, thus affecting the sperm motility and fertilization ability in these animals. As described above, further experimentation showed that Tssk4 and Odf2, a major cytoskeletal protein, are both localized in the principal piece of the sperm flagellum; it should be noted that Tssk4–Odf2 co-immunolocalization studies could not be performed because both antibodies were raised in rabbits. Based on the similar patterns of distribution in sperm flagellum, it was proposed that Tssk4 and Odf2 could interact in vivo, probably affecting the phosphorylation status of one another [56], which would in turn explain the abnormal morphology and functionality of sperm flagellum observed in the *Tssk4* knockout mice.

In 2005, Spiridonov and his colleagues described the generation of a *Tssk6* null mouse model [52]. In this publication, the authors reported that, as in the case for *Tssk1–Tssk2* null mice, *Tssk6* knockout male mice were infertile due to aberrant sperm morphology and motility, with no notable somatic abnormalities seen otherwise. The most pronounced anomaly was found in sperm head morphology and improper attachment of the sperm midpiece. Furthermore, they indicated a DNA condensation abnormality in elongating spermatids and involvement of Tssk6 in phosphorylation of histones in vitro*,* therefore proposing a role for Tssk6 in sperm chromatin remodeling. Our laboratory further analyzed the reproductive phenotype of *Tssk6*-null mice and encountered a variety of defects including low sperm number, sperm morphological defects, impaired sperm motility, and inability of sperm to fertilize in vitro [49]. Contrary to sperm from other sterile mouse knockout models (e.g., SLO3−/− [63]; CatSper1−/− [64]), *Tssk6*-null sperm were unable to fuse to zona-free eggs due to defects in Izumo1 translocation during the acrosome reaction [65, 49, 66]. Despite these findings, Tssk6-null sperm induced egg activation when the sperm–egg fusion step was bypassed by ICSI. Defects on Izumo1 translocation are consistent with defects in actin polymerization occurring in the sperm head, which we found compromised in the absence of Tssk6. In addition to the mature sperm phenotype, Tssk6 was shown to be crucial for production of histone gammaH2AX, removal of histones H3 and H4, and proper processing of protamines in mouse spermatids [67]. In this context, BRWD1, a bromodomain-containing protein, has proven to be essential for haploid gene expression and proper transcription in postmeiotic spermatids. *BRWD1* knockout mice are infertile, with notable anomalies in sperm morphology and motility, and *Tssk6* was recently found among the most significantly downregulated genes in BRDW1-deficient mouse testes during spermatogenesis [68]. Altogether, these findings strongly support a fundamental role of Tssk6 in sperm DNA condensation and chromatin remodeling, specifically pointing to the histone-to-protamine transition required for proper mouse spermiogenesis and production of functional sperm.

The *Tssk1/Tssk2* double knockout mouse model has provided very strong evidence on the relevance of these kinases in male fertility. However, characterization of the sterile phenotype has been more difficult to evaluate since the effects observed could be due to either gene, independently or as a combined effect. Current efforts in our laboratory are aimed to develop a single *Tssk1* KO and a single *Tssk2* KO mouse model and to validate these kinases as targets for the design of non-hormonal male contraceptives.

Alternatively, our analysis of the TSSK protein family predicts different ways the activity of this family of kinases could be intervened by small-molecule inhibitors. From the drug design standpoint, a variety of methods has been successfully used to design kinase small-molecule inhibitors, ranging from HTS to structure-based drug design. Strategies such as covalent modification using electrophiles targeted to conserved Cys residues accessible to the kinase ATP-binding pocket have helped in the search for specific and potent TSSK1 inhibitors [55]. According to our analysis, TSSKs would be amenable to a chemical–genetic switch for the effective validation of TSSKs as male contraception targets. This approach, described by Bishop and colleagues, includes mutation of a “gatekeeper” residue present in the ATP-binding pocket of the kinase, conferring specific sensitivity to this small-molecule inhibitor [69]. Based on this observation, our TSSK sequence analysis predicts that the gatekeeper residue in TSSKs is Met90 [70]. Furthermore, we have homology-modeled TSSK6 after the AMPK family member kinase SNF1 (PDB id 2hf9), where mutation of the gatekeeper residue Met90 to a non-bulky residue such as Gly is predicted to open up a hydrophobic pocket that would accommodate hydrophobic moieties in a pre-designed small-molecule inhibitor to be used for target validation. We propose that inhibitor-sensitive alleles of TSSKs obtained by this method could later be introduced in mice by a knock-in strategy, providing a powerful platform for in vivo validation of TSSKs as male contraception targets.

## Clinical relevance of TSSK kinases

In both mice and humans, TSSK expression appears to be restricted to the testis. In human cancer cells, however, members of this kinase family have been identified in vitro. Moreover, TSSKs have been proposed to play a role in cancer cell survival and proliferation. In this context, three separate laboratories have reported TSSK2, TSSK3, and TSSK6 in different cancer types. Using a library of activated kinases in HAE1 cells, an experimental model of human cell transformation derived from immortalized human embryonic kidney (HEK) cells [71], the first report indicated that the co-activation of MAPK and PI3K pathways could replace H-RAS^V12^ as the transformative factor in human cell lines, rendering them tumorigenic [72]. In these cells, co-expression of MEK^DD^ (constitutively active MEK1 mutant allele) and myr-AKT1 most closely mimicked the tumorigenic effect induced by H-RAS^V12^, hence providing an experimental model in search for novel transforming genes. TSSK6 was identified as one of four kinases able to cooperate with activated MEK1 to replace the transformative potential of activated AKT1 induced by H-RAS^V12^ in HEK cells. When these kinases where further analyzed in human cancers, no evidence was found on amplification of the *TSSK6* locus, although its transformative effect would categorize it as a putative oncogene, and it warrants further investigation. The second group, using a shRNA loss-of-function screening system across various human cell types, identified TSSK2 among a few kinases which were rate-limiting in promoting cell proliferation and survival, where TSSK2 was one of the 58 hits (50% cut-off or higher) that blocked cell proliferation and survival in HELA cells [73]. As an extension of this screening analysis, it was also found that RKO, a colorectal carcinoma cell line, required the expression of TSSK2 among those kinases essential for cell survival, as long as the RKOs did not express the HPV16-E7 protein [74]. Since the E7 oncoprotein is known to mediate the inactivation of p53-dependent and pRb-dependent tumor suppression, the finding that TSSK2 was dispensable for cell survival only when expression of the E7 oncoprotein was induced in RKO carcinoma cells is very significant, suggesting that TSSK2 may represent one of the essential regulators of pRb tumor suppression activity in cancer cells.

Poly-ADP-ribose-polymerase-1 (PARP) is a nuclear enzyme involved in DNA repair. PARP inhibitors are highly lethal to cells with deficiency in proteins involved in the homologous recombination pathway such as BRCA1 and BRCA2 tumor suppressors and are currently part of clinical trials for cancer therapy. Turner and colleagues, using a synthetic lethal siRNA screen, identified TSSK3 among the 779 targeted kinases that mediate sensitivity to PARP inhibitors [75]. In this study, downregulation of TSSK3 by siRNA increased the sensitivity to the PARP inhibitor in CAL51 cells, a diploid, p53-wild-type breast cancer cell line. This study is very promising since inhibition of TSSK3 through either siRNA or small-molecule inhibitors could potentially synergize the effect of treatments with PARP inhibitors, lowering their toxic effects and increasing their efficiency in *BRCA* mutation carriers.

Evaluation of *Tssk6* mRNA transcript levels as a predictor of bull sperm fertilizing ability has been proposed for use in the veterinary clinic [34]. In this work, the authors found that transcripts encoding for *TSSK6* in bull ejaculates were associated with highly motile sperm and were able to correlate these differences to parameters currently used as predictors of sperm fertilizing ability.

Finally, analysis of single-nucleotide polymorphisms (SNPs) in TSSK2, TSSK4, and TSSK6 has indicated an association with azoospermia and severe oligospermia [33, 76, 77]. In particular, the study by Su and colleagues suggests that the variant SNPs could affect the normal outcome of TSSK4 splicing process, supporting the idea that inactive splicing variants of TSSK4 could act as regulators by controlling the accessibility to kinase substrates by the active form of the kinase. Furthermore, two multicenter, randomized, controlled clinical trials and a meta-analysis have proven the clinical efficacy of Qilin pills (QLPs), a traditional Chinese medicine, for the treatment of idiopathic oligoasthenospermia [78, 79]. Using a rat model of oligoasthenospermia, a very recent study by Zhang and colleagues has demonstrated that treatment with QLPs promotes a significant improvement in male reproductive parameters, including spermatogenesis. In this study, testis expression of Tssk2, both at mRNA and protein levels, was decreased in oligoasthenospermia-induced male rats and restored by treatment with QLPs [80]. Overall, analysis of TSSK deficiencies in infertile men might result in improved therapies or diagnostic tools for male infertility.

## TSSK kinases as therapeutic targets for male contraception

Considering proteins in the TSSK kinase family as candidate targets for drug design of non-hormonal male contraceptives, five main criteria are to be taken into consideration, as follows: (1) functional criteria by which the target gene is required for spermatogenesis and/or sperm function and where targeted gene disruption leads to a male infertile phenotype; (2) specific expression criteria where the target should be expressed in a testis-specific manner and preferably expressed postmeiotically in germ cells; (3) applied investigation criteria where the target activity should allow for a rational design of candidate inhibitor drugs; (4) safety criteria by which inhibition of the target should have no secondary physiological effects; and (5) reversibility criteria by which, after cessation of treatment, sperm production returns to normal values that are compatible with normal fertility. As described above, a number of studies by several laboratories have provided evidence supporting both the functional and specific expression criteria for using TSSK kinases as a potential target for drug design of a male contraceptive pill.

Toward fulfilling the third criteria, during the last few years, our group and others have been actively working toward the development of an in vitro assay, amenable for HTS of TSSK kinase inhibitors. In 2016, the group led by John Herr reported the production of full-length enzymatically active, recombinant TSSK2 using a baculovirus expression system. Furthermore, TSSK2 kinase activity was successfully tested by a mobility shift assay with bacterially produced TSKS (isoform 2) and casein as substrates [81]. To expand this knowledge, Hawkinson and colleagues applied a HTS screen of around 17,000 compounds using a mobility shift assay with recombinant TSSK2 and fluorescently labeled glycogen synthase (5-FAM-GS) as substrate in a 384-well plate format. These authors were able to identify two, highly potent series of ATP-site TSSK2 inhibitors with either a pyrrolopyrimidine or pyrimidine core [82]. Three pyrrolopyrimidine core compounds included in this study, with highest potency against TSSK2, had been reported to be also effective inhibitors of several other kinases including IGF-1. On the other hand, among the hits found in the initial screen, TAE684, a pyrimidine core inhibitor, was chosen for further evaluation and several of its analogs tested against TSSK2. Based on the two most potent TSSK2 inhibitors in this series (Compounds 17 and 18), Compound 19 was newly synthesized and submitted for kinome profiling (Reaction Biology Corporation, Malvern, PA) against 369 wild-type kinases. Interestingly, Compound 19 showed a broad range of inhibition potencies for TSSK family members (TSSK1 > TSSK2 > TSSK3 > TSSK6), with the highest potency being against TSSK1 and TSSK2, pointing to a correlation between selectivity and sequence homology. Overall, these newly described TSSK inhibitors may prove useful tools for lead optimization by medicinal chemistry, as well as crystallography analysis of TSSK1 and TSSK2 with ligands bound to their ATP-binding sites. Alternatively, identification of TSSK allosteric inhibitors may lead to more selective and potent small-molecule inhibitors of the TSSK kinases family.

## Summary

Several laboratories are actively engaged in discovery and validation of non-hormonal male contraceptives targets. The unique characteristics of the TSSK protein kinases make them very good candidates as potential targets for development of non-hormonal male contraceptives, as follows: (1) protein kinases have become very attractive targets for development of novel drugs; (2) the sterile phenotypes of the *TSSK6* and the double *TSSK1/TSSK2* knockout mice strongly indicate that this kinase family plays an essential role in reproduction; (3) the sequences of TSSK family members and their testis-specific expression are evolutionary conserved in vertebrates and invertebrates; (4) this group of kinases is developmentally regulated during spermatogenesis and is only expressed postmeiotically in germ cells, suggesting that inhibition of TSSK kinases activity with a specific drug could inhibit spermatogenesis and/or fertilization in a reversible fashion; and (5) unique features of the TSSK family supports the likelihood of finding specific small-molecule inhibitors. As part of a multicenter collaborative effort, ongoing research in our laboratory is focused on the already promising search for an effective, cell-permeable inhibitor against TSSKs that may lead to a marketable male contraceptive pill.
